# ﻿New psychropotid species (Echinodermata, Holothuroidea, Elasipodida) of the Western Pacific with phylogenetic analyses

**DOI:** 10.3897/zookeys.1088.69141

**Published:** 2022-03-09

**Authors:** Chuan Yu, Dongsheng Zhang, Ruiyan Zhang, Chunsheng Wang

**Affiliations:** 1 School of Oceanography, Shanghai Jiao Tong University, Shanghai, China Shanghai Jiao Tong University Shanghai China; 2 Key Laboratory of Marine Ecosystem Dynamics, Second Institute of Oceanography, Ministry of Natural Resources, Hangzhou, China Key Laboratory of Marine Ecosystem Dynamics, Second Institute of Oceanography, Ministry of Natural Resources Hangzhou China; 3 Southern Marine Science and Engineering Guangdong Laboratory (Zhuhai), Zhuhai, Guangdong, China Southern Marine Science and Engineering Guangdong Laboratory (Zhuhai) Zhuhai China; 4 State Key Laboratory of Satellite Ocean Environment Dynamics, Ministry of Natural Resources, Hangzhou, China State Key Laboratory of Satellite Ocean Environment Dynamics, Ministry of Natural Resources Hangzhou China

**Keywords:** *Benthodytes*, deep-sea, holothurians, *Psychropotes*, taxonomy

## Abstract

Holothurians of the family Psychropotidae are widely distributed but remain the least studied deep-sea holothurians. On an expedition to the Western Pacific, six psychropotid specimens were collected by the Jiaolong Human Operated Vehicle (HOV). Through morphological examination, four of them were identified as a new species, *Benthodytesjiaolongi***sp. nov.**, which was characterized as having minute papillae, a narrow brim, and a terminal anus; and the ossicles were rods and primary crosses. The remaining two specimens were identified as *Psychropotesverrucicaudatus* Xiao, Gong, Kou & Li, 2019, first recorded at the Kyushu-Palau Ridge. The phylogenetic analysis showed that *B.jiaolongi***sp. nov.** and *P.verrucicaudatus* were embedded in the clades *Benthodytes* and *Psycheotrephes*, respectively, and that *Benthodytes* was paraphyletic. The new species clustered with *Benthodytessanguinolenta* and was separated from the clade containing the other *Benthodytes* species.

## ﻿Introduction

Holothurians of the family Psychropotidae (Elasipodida) were first identified by Théel (1882) who defined four genera of deep-sea sea cucumbers discovered on the H.M.S. Challenger Expedition. Subsequently, Hérouard (1909) and Belyaev and Vinogradov (1969) erected *Triconus* Hérouard and *Nectothuria* Belyaev & Vinogradov, which were later regarded as synonyms of *Psychropotes* by Hansen (1975). Meanwhile, *Euphronides* Théel, 1882 was also accepted as a synonym of *Psychropotes*. Psychropotidae comprises three genera and 37 species. Hansen (1975) distinguished the three genera by the presence or absence of an unpaired dorsal appendage, the position of the anus, and the presence or absence of circum-oral (or post-oral) papillae. Although, taxonomists have long worked on this family, Psychropotidae are still the least studied deep-sea holothurians. Thus, the phylogenetic relationships within Psychropotidae remain unclear.

**Figure 1.**
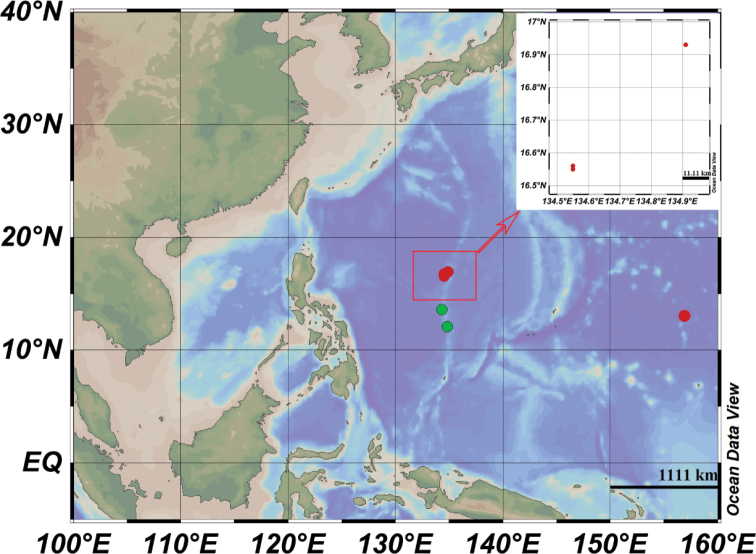
Red dots show the location of *Benthodytesjiaolongi* sp. nov. and green dots indicate the location of *Psychropotesverrucicaudatus* Xiao, Gong, Kou & Li, 2019.

An expedition of the Jiaolong Human Operated Vehicle (HOV) concentrated on further increasing our understanding of the biodiversity, connectivity, and conservation value of the Western Pacific. During sampling, six specimens of Psychropotidae were collected from seamounts on the Kyushu-Palau Ridge and Weijia Guyot (Fig. 1). Based on an analysis of the external morphological characters and ossicles, we identified four specimens as a new species (*Benthodytesjiaolongi* sp. nov.) and the other two as new records of *Psychropotesverrucicaudatus* Xiao, Gong, Kou & Li, 2019.

## ﻿Materials and methods

### ﻿Sampling and morphological observations

The samples described in the present study were collected by the Jiaolong HOV at a depth of 2408–2602 m, from the Kyushu-Palau Ridge and Weijia Guyot. Before preservation, a Canon EOS 5DII camera (Canon Inc., Tokyo, Japan) was used to take photographs of the specimens on board the ship. Then, a piece of dorsal tissue was cut from all specimens and frozen at -20 °C for DNA extraction. Finally, the specimens were fixed in 10% seawater formalin or 99% alcohol and deposited at the Repository of Second Institute of Oceanography (RSIO). Sodium hypochlorite was used to dissolve body tissues (tentacles, dorsum, ventrum, brim, dorsal warts and gonads), and ossicles present in these tissues were rinsed five times with purified water. The ossicles were observed using a scanning electron microscope (TM 1000; Hitachi, Ltd., Tokyo, Japan).

### ﻿PCR amplification and phylogenetic analysis

Total genomic DNA was extracted from 100 mg of muscle tissue using a DNeasy Blood & Tissue Kit (QIAGEN, Hilden, Germany) according to the manufacturer’s instructions. Two partial mitochondrial genes, 16S rRNA and cytochrome oxidase subunit 1 (COI), were amplified using primers 16S-arL/brH and COI-ef/er (Miller et al. 2017). The PCR reactions were performed using a 50-µL reagent mix, containing 25 μL 2× Phanta Max Master Mix (Vazyme, Biotech Co., Ltd., Nanjing, China), 20-μL DNase free ddH_2_O, 2-μL of each primer, and 1-μL template DNA, as suggested by the manufacturer. The PCR amplification procedure is shown in Table 1. PCR products were confirmed by 1.5% agarose gel electrophoresis and purified using an OMEGA PCR kit (Omega, Biotek, Norcross). The purified PCR products were sequenced on an ABI 3730XL sequencer (Sangon, Biotech Co., Ltd., Shanghai). Sequence data were edited with Geneious R6.1.6 (Matthew et al. 2012) and deposited in GenBank (Table 2).

**Table 1.** PCR amplification procedures.

Primer	Sequence 5′→ 3'	PCR procedure
COI-ef	ATAATGATAGGAGGRTTTGG	Pre denaturation: 95 °C for 3 min
COI-er	GCTCGTGTRTCTACRTCCAT	40 cycles:
		Denaturation: 95 °C for 40 s
		Annealing: 45 °C for 40 s
		Extension: 72 °C for 50 s
16S-arL	CGCCGTTTATCAAAAACAT	Pre denaturation:95 °C for 3 min
16S-brH	CCGGTCTGAACTCAGATCACG	35 cycles:
		Denaturation: 95 °C for 40 s
		Annealing: 50 °C for 40 s
		Extension: 68 °C for 50 s

For a more comprehensive phylogenetic analysis, we not only used the sequences of Psychropotidae obtained here, but also used mitochondrial sequences of Elpidiidae Théel, 1882 and two species of Stichopodidae Haeckel, 1886, an outgroup (Table 2). Twenty-five COI and 18 16S sequences were aligned using MAFFT 7 (Katoh and Standley 2013) using the E-INS-I strategy. Alignment gaps and missing data were represented as ‘-’ and ‘?’. The 16S and COI alignments were concatenated (COI/16S = 687/578 bp), analyzed with Maximum likelihood (ML) and Bayesian inference (BI) algorithms. JModelTest 2.1.10 (Darriba et al. 2012) was used to find the best-fit model from 88 competing models using Akaike information criterion (AIC) calculations. In each case, GTR+I+G was the best-fit model for BI analyses. MrBayes 3.2 (Huelsenbeck and Ronquist 2001) was used to conduct BI analyses. Markov Chain Monte Carlo (MCMC) iterations were run for 1 000 000 generations with sampling every 100 generations. The first 25% of trees were discarded as burn-in, and the consensus trees were summarized in 75% majority-rule trees. RAxML GUI 1.5 (Silvestro and Michalak 2012; Stamatakis 2014) was used to perform the ML analysis with the GTR+GAMMA+I substitution model for 1000 bootstraps, as recommended by Miller et al. (2017).

**Table 2.** Details of specimens and GenBank accession numbers in this study.

Family	Species	GenBank accession number	
		*16S*	*COI*
Psychropotidae Théel, 1882	*Benthodytesmanusensis* Xiao, Li & Sha, 2018	MH627223.1	MH627222.1
	*Benthodytessanguinolenta* Théel, 1882		HM196507.1
	*Benthodytesmarianensis* Li, Xiao, Zhang & Zhang, 2018	MH049433.1	MH049435.1
	*Benthodytesjiaolongi* sp. nov.	MW992746	MW990356
	*Benthodytesjiaolongi* sp. nov.	MW992747	MW990357
	*Psycheotrephesexigua* Théel, 1882		KX874392.1
	*Psychropoteslongicauda* Théel, 1882	DQ777099.1	KU987469.1
	*Psychropotesmoskalevi* Gebruk & Kremenetskaia in Gebruk et al., 2020	MN310400.1	MN313655.1
	*Psychropotesraripes* Ludwig, 1893	MN310403.1	MN313656.1
	*Psychropotesverrucicaudatus* Xiao, Gong, Kou & Li, 2019	MW992749	MW980089
	*Psychropotesverrucicaudatus* Xiao, Gong, Kou & Li, 2019	MW992748	MW980088
Elpidiidae Théel, 1882	*Peniagonediaphana* Théel, 1882	KX856725.1	KX874384.1
	*Peniagoneincerta* Théel, 1882		HM196402.1
	*Peniagone* sp. AKM-2016	KX856726.1	KX874385.1
	*Peniagonevignoni* Hérouard, 1901		HM196381.1
	*Elpidiaglacialis* Théel, 1876		HM196413.1
	*Amperimarobusta* Théel, 1882	KX856728.1	KX874381.1
	*Protelpidiamurrayi* Théel, 1879	KX856727.1	KX874382.1
	*Scotoplanes* sp.TT-2017		LC230158.1
Laetmogoidae Ekman, 1926	*Laetmogonewyvillethomsoni* Théel, 1879		HM196504.1
	*Pannychiamoseleyi* Théel, 1882	KX856731.1	KX874380.1
	*Benthogoneabstrusa* Sluiter, 1901	KX856733.1	KX874374.1
	*Enypniasteseximia* Théel, 1882		
Pelagothuriide Ludwig, 1893		KX856730.1	KX874383.1
Stichopodidae Haeckel, 1896	*Apostichopuscalifornicus* Stimpson, 1857	KP398509.1	KP398509.1
	*Apostichopusparvimensis* H.L. Clark, 1913	KX856750.1	KX874373.1

## ﻿Results and discussion

### ﻿Order Elasipodida Théel, 1882

**Suborder Psychropotina Hansen, 1975**

**Family Psychropotidae Théel, 1882**

#### ﻿Genus Benthodytes

Taxon classificationAnimaliaElasipodidaPsychropotidae

Théel, 1882

6628A0F2-FF02-5656-8D47-9090EAD15C4A

##### Diagnosis

**(according to Hansen 1975).** Anus dorsal. Unpaired dorsal appendages absent. Circum-oral (or post-oral) papillae present. Tentacles soft, pliable, and retractile.

#### ﻿ Benthodytes jiaolongi sp. nov.

Taxon classificationAnimaliaElasipodidaPsychropotidae

4C5D41F2-E001-5F16-9CC4-C7BD5818D277

http://zoobank.org/85760628-2F68-4800-B9DA-694C8BF167A2

Figs 2 , 3 , 4

##### Type material examined.

***Holotype***: RSIO6017101, adult specimen, collection number: DY60-JL171-B01, 16.935°N, 134.911°E,12 January 2021, 2602 m; ***Paratype***: RSIO3710601, adult specimen, collection number: DY37-JL106-B01, 13.017°N, 156.947°E, 1 May 2016, 2408 m.

##### Non-type material examined.

RSIO590504, adult specimen, collection number: DY59-ROV05-B04, 16.916°N, 134.916°E, 20 July 2020, 2692 m; RSIO590506, adult specimen, collection number: DY59-ROV05-B06, 16.933°N, 134.916°E, 20 July 2020, 2453 m.

##### Diagnosis.

Body elongated and subcylindrical when fixed. Skin red with violet, thin, soft. No obvious large papillae arranged on dorsal surface. Some minute papillae, conical with tips, on the anterior dorsum. Brim narrow, thin, flattened. Mouth ventral, anus terminal. Eighteen tentacles; circum-oral papillae present. Dorsal ossicles include rods and primary crosses with four arms. Rods present in tentacles. Ossicles of ventrum not observed.

##### Description of holotype.

**Figure 2.**
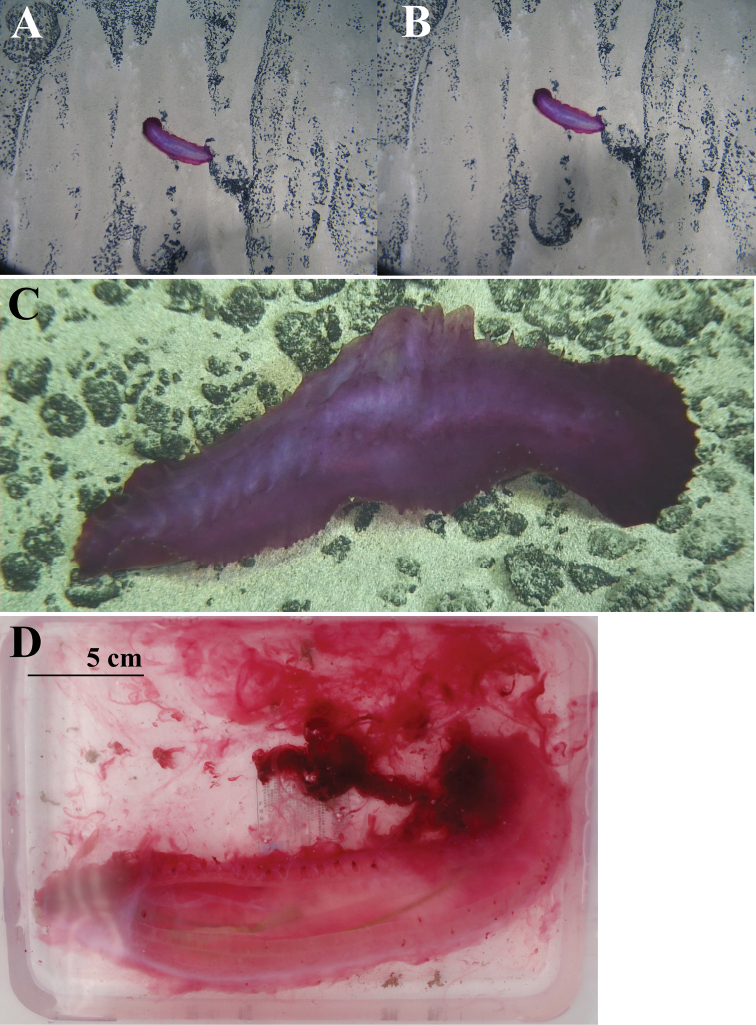
**A, B***Benthodytesjiaolongi* sp. nov. (RSIO3710601, holotype) in situ on the seamount Weijia Guyot **C** specimen (RSIO6017101, paratype) in situ on the Kyushu-Palau Ridge **D** specimen (paratype) before preservation in 10% seawater formalin.

(RSIO6017101). Length was approximately 25 cm before preservation in 10% seawater formalin. Color violet in life (Fig. 2C); skin transparent, thin, soft, and gelatinous after fixing. Brim retracted less than 0.7 cm in width. Approximately nineteen pairs of dorsal papillae poorly developed, minute, closely placed in two bands along anterior dorsal radii. Another four single minute papillae on posterior dorsal edge. Approximately 28 pairs midventral tube feet arranged in two rows. Mouth ventral, with circum-oral papillae. Anus terminal, unguarded. Due to the contraction, tentacles could not be clearly observed. Few ossicles observed. Dorsal ossicles in the anterior body wall, consisting of primary crosses with spiny arms, and spinous rods (Fig. 4A–F). Rods approximately 400 μm long, arms of crosses approximately 200 μm long. Tentacles with rods, 400–500 μm long (Fig. 4G–J). Other body parts devoid of ossicles.

##### Description of paratypes.

RSIO3710601. Specimen approximately 22 cm in length, 5 cm wide at maximum point. Color red-violet *in situ* at the seabed (Fig. 2A, B); pale violet at sea surface, with transparent skin; white color after preservation in 10% seawater formalin for 5 years. Paired dorsal papillae as present in holotype absent, minute papillae also not distinguished. Owing to long-term preservation, quantity of midventral tube feet could not be determined, but were arranged in two rows. Brim could not be distinguished. Mouth ventral, with circum-oral papillae, anus terminal. Eighteen tentacles retracted to stalk. Ossicles not observed.

**Figure 3.**
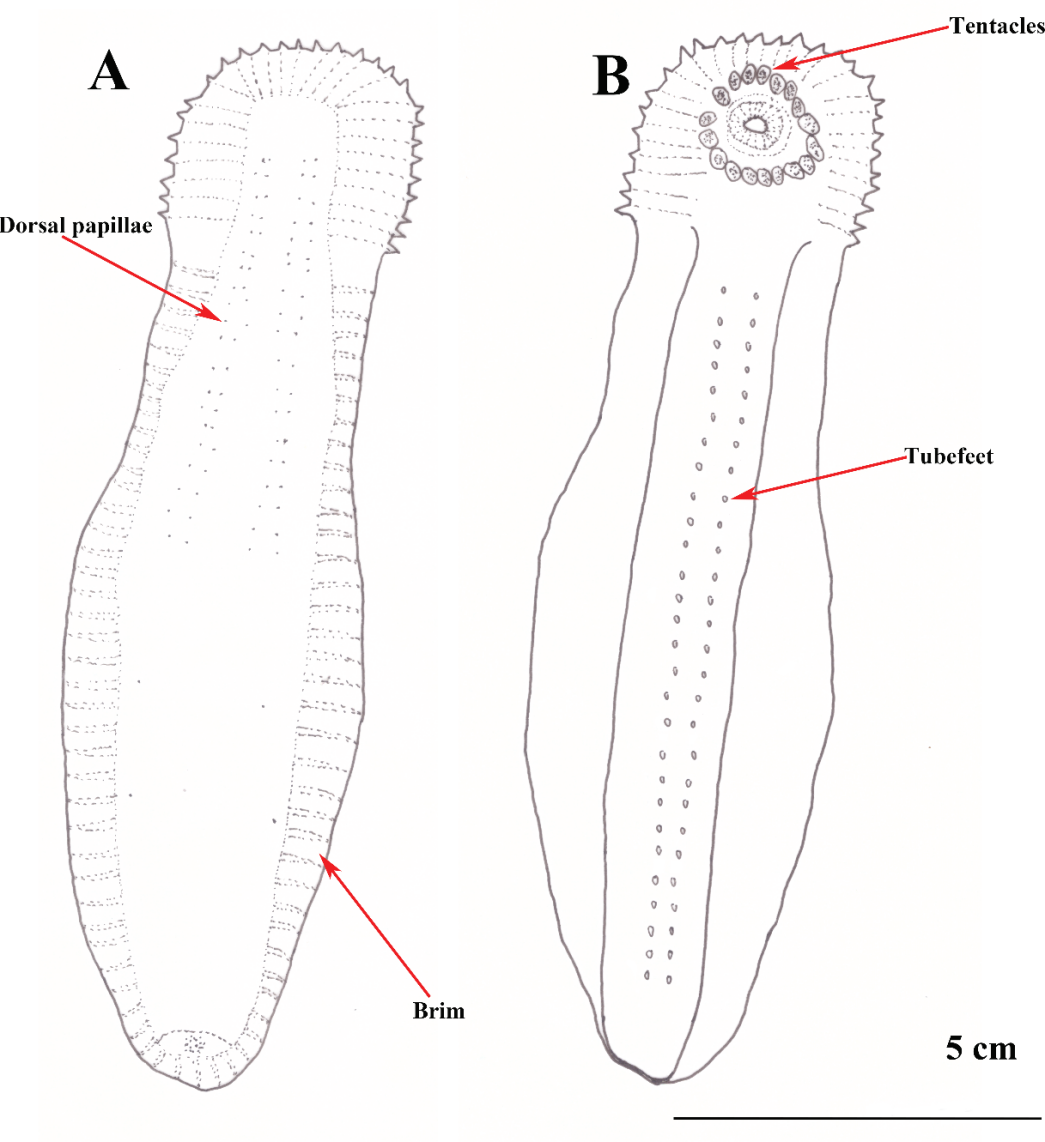
*Benthodytesjiaolongi* sp. nov. **A** dorsal view **B** ventral view.

RSIO590504.Specimen approximately 22 cm in length before preservation in 10% seawater formalin. Color red-violet on deck, skin transparent; white color after preservation. During sampling, a piece of sponge was stuck in the ROV pump sampler, and the specimen was damaged by the sponge, meaning that the tentacles could not be determined and the dorsal tips could not be distinguished. Quantity of midventral tube feet could not be determined. Mouth ventral, anus terminal. Ossicles not observed.

**Figure 4.**
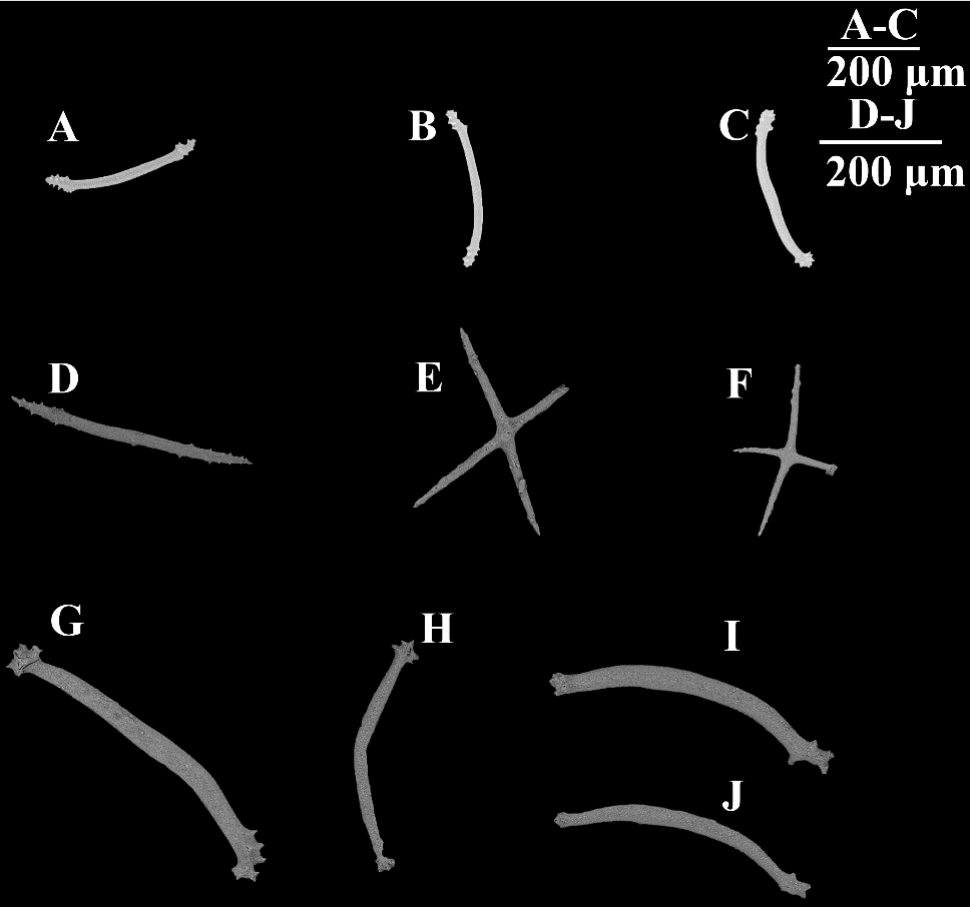
**A–C** scanning electron micrographs of dorsal body wall ossicles from *Benthodytesjiaolongi* sp. nov., RSIO590506 **D–F** dorsal body wall ossicles from *Benthodytesjiaolongi* sp. nov., RSIO6017101 **G–J** ossicles of tentacles.

RSIO590506. Specimen approximately 13 cm in length before preservation in 99% alcohol and heavily damaged. Color red-violet at sea surface, skin transparent. The specimen was stained with sponge as was RSIO590504 and many external characters could not be distinguished. Mouth ventral, anus terminal. Few rods observed on dorsal region (Fig. 4A-C). Rods approximately 400 μm, spine terminal. Ossicles from body wall not observed.

##### Etymology.

The name is derived from the first Chinese HOV ‘Jiaolong’.

##### Type species.

*Benthodytestypica* Théel, 1882 (by original designation).

##### Type locality.

Kyushu-Palau Ridge, tropical Western Pacific. Depth: 2453–2692 m.

##### Distribution.

Known from Weijia Guyot and Kyushu-Palau Ridge.

##### Remarks.

Hansen (1975) revised the genus *Benthodytes* and proposed that this genus, except *Benthodytessuperba* Koehler & Vaney, 1905, could be divided into two distinct groups based on the ossicles and external morphology.

The first group was characterized by the regular crosses, ossicles with bipartite central apophysis and well-developed dorsal papillae. This group included five species: *B.incerta* Ludwig, 1894; *B.lingua* Perrier, 1896; *B.valdiviae* Hansen, 1975; *B.sibogae* Sluiter, 1901a and *B.plana* Hansen, 1975. *Benthodytessanguinolenta* Théel, 1882 and *B.typica* Théel, 1882 formed the second group characterized by strongly reduced rod ossicles, and minute dorsal papillae.

Recently, five more species were identified: *B.gosarsi* Gebruk, 2008; *B.wolffi* Rogacheva & Cross in Rogacheva et al. 2009; *B.violeta* Martinez, SolísMarín & Penchaszadeh, 2014; *B.manusensis*Xiao et al., 2018; *B.marianensis*Li et al., 2018. They can be assigned to first group.

*Benthodytesjiaolongi* sp. nov. clearly belongs in the genus *Benthodytes* and is close to *Benthodytessanguinolenta* Théel, 1882 and *Benthodytestypica* Théel, 1882, for the minute papillae and reduced rod ossicles.

*Benthodytestypica* was described by Théel in 1882 based on specimens collected by the *Challenger* Expedition. The original description indicated approximately eight, minute, retractile processes located on each of the dorsal ambulacra and unbranched spinose calcareous spicula scattered on the integument. Hansen (1975) re-examined *B.typica* and reported that the specimens showed considerable variation. *Benthodytespapillifera* Théel, 1882 was described based on 13 specimens taken from three Pacific Challenger stations. Théel (1882) described this species as being similar to *B.sanguinolenta* based on the tentacles and tube feet. Hansen (1975) re-examined specimens from each of the stations and proposed that the variation in *B.papillifera* represented the geographic variation of *B.typica*. In the original description of *Benthodytesglutinosa* Perrier, 1896, Perrier (1896) indicated that the differences from *B.typica* were the more elongated shape and the complete absence of dorsal papillae. Hansen (1975) considered this species to be a synonym of *B.typica*.

In general, the morphological features of *B.typica* can be summarized as follows: 3–7 pairs of minute papillae arranged on the dorsal surface and rods scattered on the body integument and tentacles. *Benthodytesjiaolongi* sp. nov. differs from *B.typica* in its arrangement and number of dorsal papillae and composition of ossicles. The dorsal minute papillae of *Benthodytesjiaolongi* sp. nov. are arranged in two bands along the anterior dorsal ambulacra, and those of *B.typica* are arranged in a row with 3–7 pairs of papillae. The rods of *B.jiaolongi* sp. nov. are present in the tentacles and dorsum, and the primary crosses are only present in the dorsum. However, *B.typica* only present rods scattered on the ventrum, dorsum and tentacles.

The characteristics of *B.sanguinolenta* as described by Théel (1882) included the many minute retractile processes scattered on the dorsal surface; the form of calcareous deposits could not be distinguished. According to a re-examination by Hansen (1975), the dorsal minute papillae were arranged in two radial bands and the rods were only present on the midventral tube feet and tentacle discs of specimens from station 663. Rogacheva et al. (2009) recorded *B.sanguinolenta* and the main characteristics can be described as: minute dorsal papillae arranged in two bands or between the two bands; approximately 1–4 papillae placed in a band, narrowing to one or two papillae at the posterior end; ossicles were not found. The differences in the characteristics between the new species *B.jiaolongi* sp. nov. and *B.sanguinolenta* can be listed as follows: (1) Dorsal papillae of *B.sanguinolenta* are arranged in two bands, whereas those of the new species were arranged in two rows on the anterior dorsal ambulacra; (2) Ossicles of the new species were only present in the tentacles and in the dorsum. Rods are present in the tentacles and dorsum, and primary crosses are only present in the dorsum; whereas the rods are only present in tube feet and in the tentacles in *B.sanguinolenta*.

###### Genus *Psychropotes* Théel, 1882

#### ﻿ Psychropotes verrucicaudatus

Taxon classificationAnimaliaElasipodidaPsychropotidae

Xiao, Gong, Kou & Li, 2019

9A0938C7-C0E0-516A-9DC5-0B63FDEF6F3D

Figs 5 , 6 , 7

Psychropotes verrucicaudatus Xiao, Gong, Kou & Li, 2019: 421–430.

##### Material examined.

Catalog number: RSIO6018004, adult specimen, collection number: DY60-JL180-B04, 13.569°N, 134.352°E, 25 January 2021, 2469 m; Catalog number: RSIO6017005, adult specimen, collection number: DY60-JL170-B05, 12.079°N, 134.860°E, 8 January 2021, 2361 m.

##### Description.

**Figure 5.**
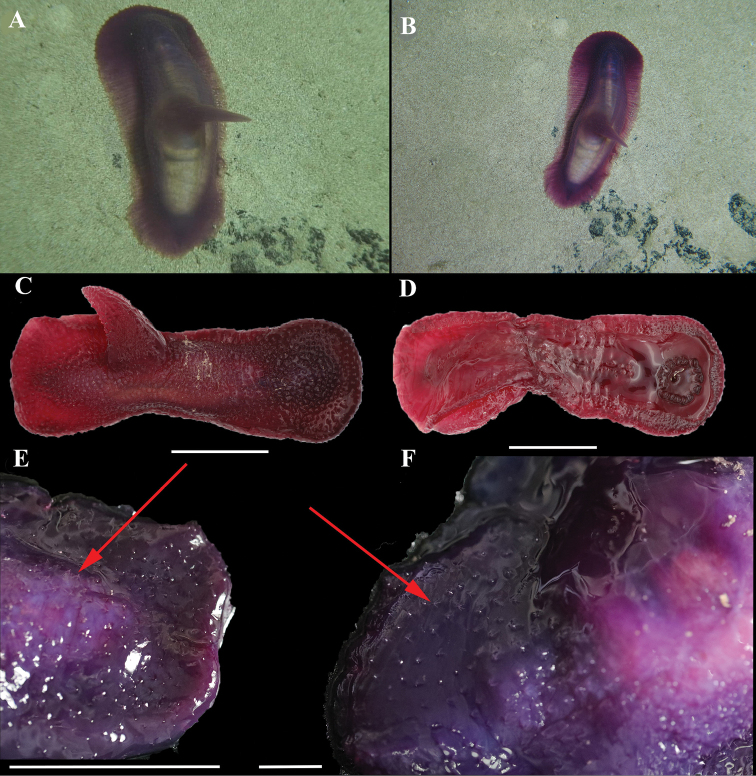
**A, B***Psychropotesverrucicaudatus* Xiao, Gong, Kou & Li, 2019 (RSIO6018004) in situ **C, D** specimen (RSIO6018004) before preservation **E, F** red arrows point to the giant ossicles, specimen (RSIO6018004) after preservation in 10% seawater formalin. Scale bars: 5 cm (**A–E**); 1 cm (**F**).

RSIO6018004. Specimen resembles a barbell after collection, approximately 20 cm in length before preservation 10% seawater formalin (Fig. 5C, D). Before preservation, height of appendage was approximately 50 mm, and width at base approximately 30 mm (Fig. 5C, D). Dorsal skin transparent with brownish red color on seabed and dark brown on deck. Warts covering dorsal skin and appendage; giant ossicles in warts visible (Fig. 5E, F). Approximately 30 pairs of degenerated tube feet arranged in two rows along middle of ventrum. Sixteen tentacles forming a circle. Brim broad and covered with warts on dorsum.

**Figure 6.**
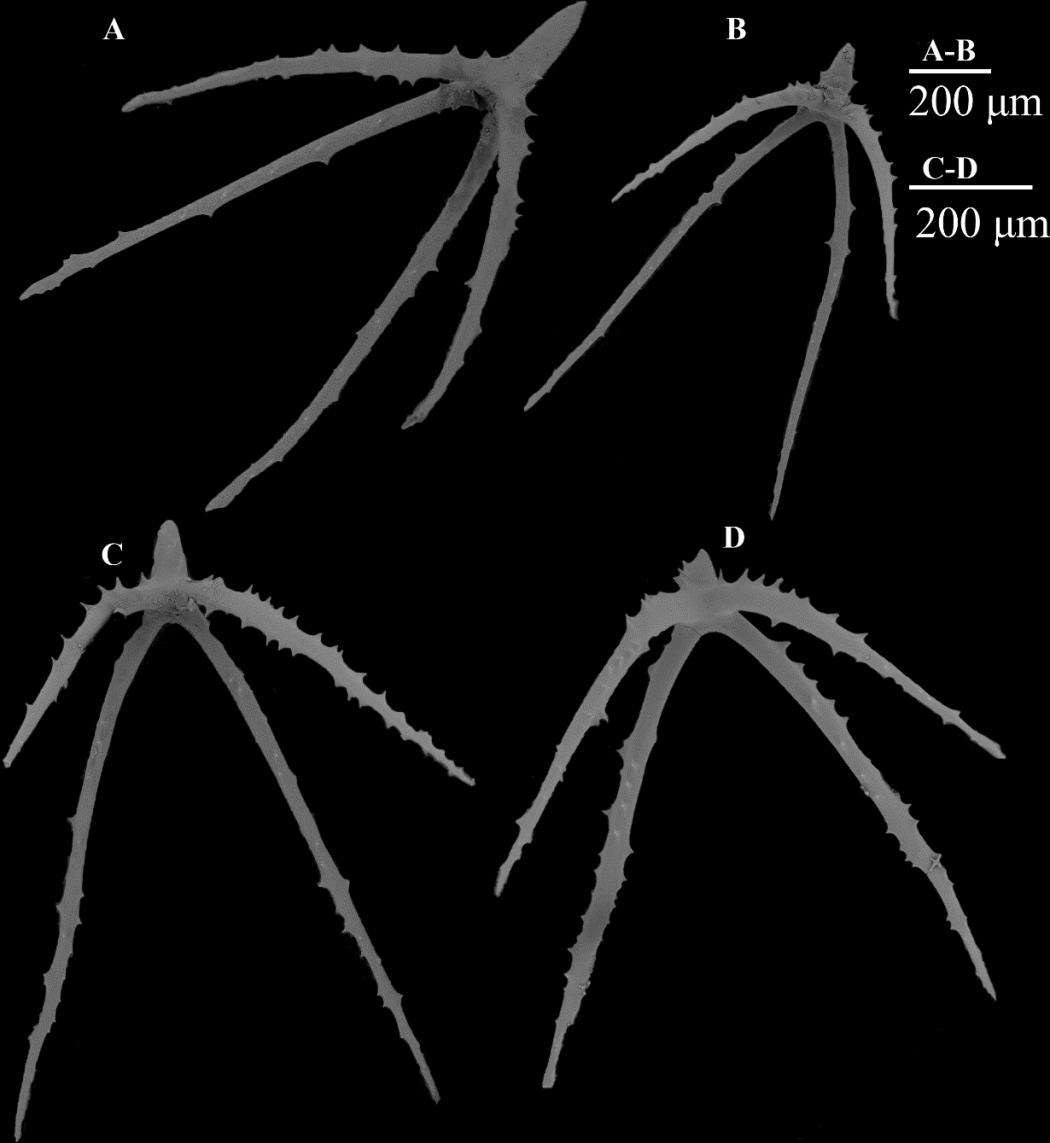
*Psychropotesverrucicaudatus***A–D** giant ossicles from the dorsal warts.

**Figure 7.**
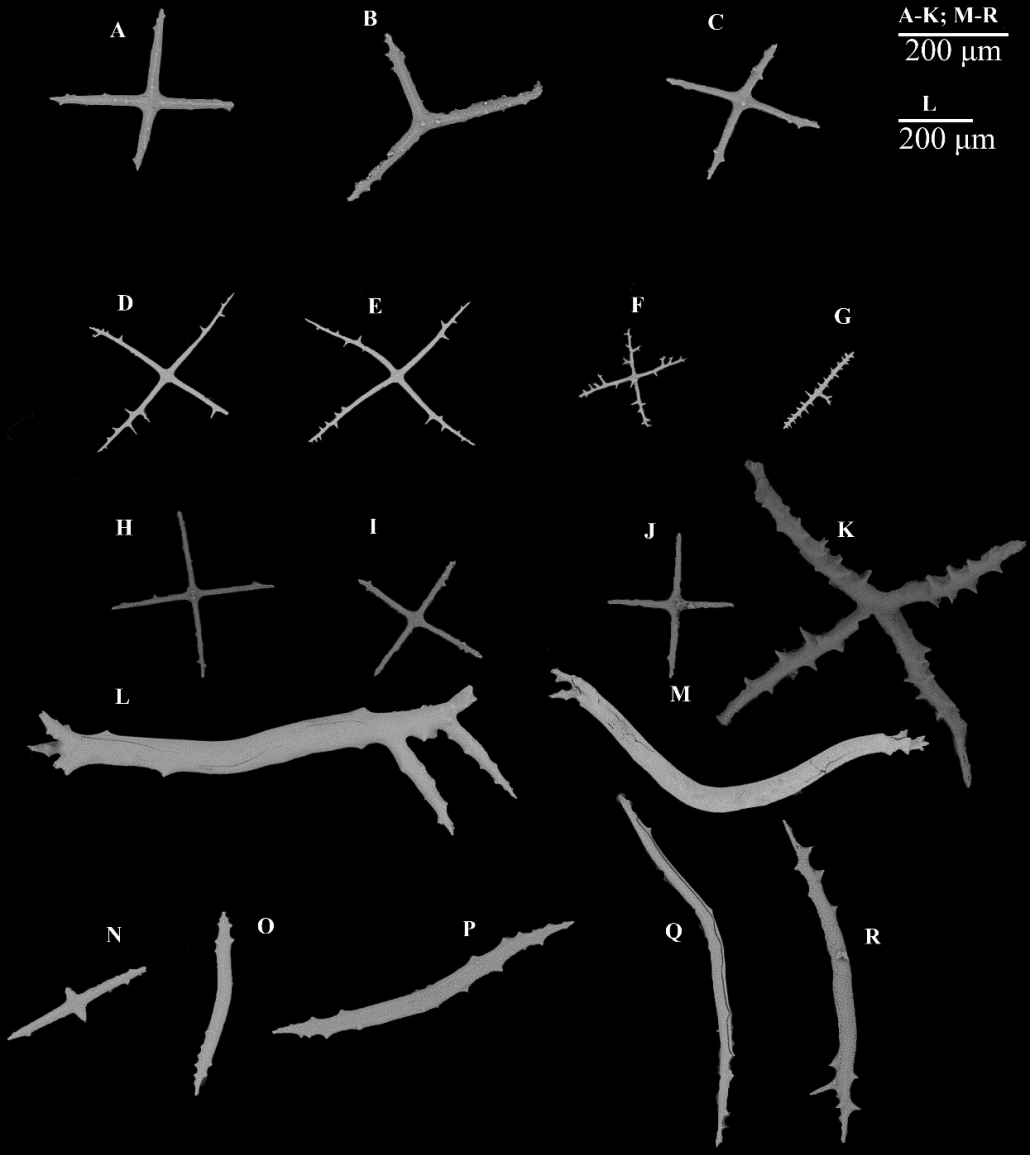
*Psychropotesverrucicaudatus* ossicle from **A–C** ventral body wall **D–G** dorsal body wall **H–K** brim **L–R** tentacle.

**Figure 8.**
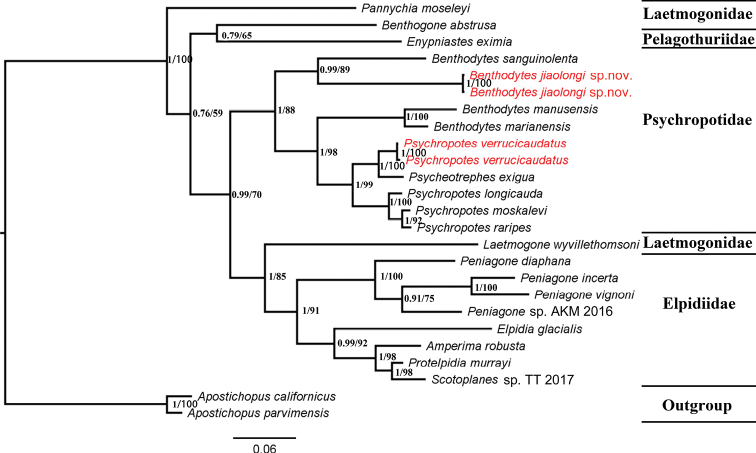
Bayesian inference (BI) and maximum likelihood (ML) trees based on the concatenated sequences. The Bayesian posterior probabilities (BI) and Maximum likelihood bootstrap (BS) values are shown as BI/ML at each node. Scale bar indicates the evolutionary branch length.

A giant cross with four arms visible in each wart. Arms 800–1000 μm in length, and maximum width between large arms approximately 500 μm. Arm flexion approximately 250 / 400 μm (Fig. 6A–D). Height of central rudimentary apophyses approximately 200–300 μm. Ventral ossicles divided into two types: primary cross with spiny arms (Fig. 8A, C) and cross with three arms (Fig. 7B), length of arm approximately 200 μm. Primary crosses with spinous arms in dorsum (Fig. 7D–F) and brim (Fig. 7H–J); arms up to 200 μm in length. Dorsal ossicles with spinous rod, 170 μm in length (Fig. 7G), and large primary crosses with spiny arms in brim (Fig. 7K). Tentacles with rods with irregular shape (Fig. 7L–R). Large rod with two apophyses at the end, approximately 900–1000 μm in length (Fig. 7L-M); small rod with apophyses in middle area was approximately 200 μm in length (Fig. 7N). Other rods with spiny arms, 500–800 μm in length (Fig. 7O-R).

RSIO6017005. Specimen approximately 18 cm in length, height of appendage approximately 40 mm, and width at base approximately 20 mm. Mouth and anus ventral. Skin transparent, light brown color. Dorsal skin and appendage covered with warts; warts also present in dorsum of brim. Giant ossicles visible in warts. Tentacles damaged, more than 12. Ossicles as in RSIO6018004.

##### Type locality.

Jiaolong Seamount, South China Sea, Western Pacific Ocean, sandy bottom, depth 3615 m.

##### Type species.

*Psychropoteslongicauda* Théel, 1882.

##### Distribution.

Known from Jiaolong Seamount of South China Sea and Kyushu-Palau Ridge.

##### Intraspecific variation.

The specimens were clearly a new record for the South China Sea, as the species was previously known only from the Jiaolong seamount. The present specimens differed from those of Xiao et al. (2019) in external morphology and the ossicles. Due to the bad preservation, Xiao et al. (2019) could not observe the ossicle assemblage of the warts, which was possible in the specimens here under study.

The intraspecific differences can be listed as follows: (1) In the present specimens, the skin was transparent and the color was darker than that of the type specimen; (2) The width of the appendage at the base was also larger than that of the type specimen; (3) The length of the primary crossing arms distributed in the dorsum, ventrum, and brim was longer than that of the type specimen. Furthermore, the spinous rod of the dorsal ossicles was not present in the type specimen, and the ventral body wall of the specimens did not possess the tripartite ossicles of the type specimens; and (4) Most of the ossicles of the tentacles in our specimens were the same as those of type specimen, but longer.

### ﻿Phylogenetic analyses

Owing to limited genetic sequences, the phylogenetic relationships of Elasipodida remains little studied. The new classification system of Elasipodida was constructed by Miller et al. (2017), whereby Deimatidae was separated from Elasipodida. The remaining families of Elasipodida included Elpidiidae, Laetmogonidae, Pelagothuriidae, and Psychropotidae, but their positions within Elasipodida remained unresolved. Li et al. (2018) used mitochondrial and nuclear genes to perform phylogenetic analyses of Elasipodida, especially the Psychropotidae, and the results showed that *Benthodytes* was a paraphyletic group of Psychropotidae based on analyses of the mitochondrial genes.

To obtain clearer phylogenetic relationships, we concatenated 25 COI and 18 16S sequences into a dataset to build ML and BI trees. Although the genetic sequences were limited, the topological structures of the ML and BI trees were mostly consistent with morphological classification. In addition, *B.jiaolongi* sp. nov. and *P.verrucicaudatus* were embedded in the clades of *Benthodytes* and *Psycheotrephes*, respectively (Fig. 8). The phylogenetic relationships of Psychropotidae clustered into four parts and were inconsistent with the traditional classification system (Hansen 1975). *Benthodytes* was divided into two clades in Psychropotidae and the new species was clustered with the clade of *B.sanguinolenta*. In addition, *Psychropotes* was a sister group to *Psycheotrephes*, and the clade of *B.jiaolongi* sp. nov. and *B.sanguinolenta* was a sister group to other Psychropotidae species. *Psychropotesverrucicaudatus* was not recovered in the clades of *Psychropotes*, but was clustered in the clade of *Psycheotrephesexigua* Théel, 1882, which meant that *P.verrucicaudatus* might belong to *Psychrotrephes*. Elpidiidae clustered into two clades: (1) *Penigone* clustered together into a supported group, but *Peniagonediaphana* Théel, 1882 was a sister group to other *Peniagone* species; (2) The other four genera of Elpidiidae clustered into a group, and *Elpidiaglacialis* Théel, 1876 was distant from the other three genera. *Protelpidiamurrayi* Théel, 1879 and *Scotoplanes* sp. TT 2017 were sister taxa, and *Amperimarobusta* Théel, 1882 was sister to these genera.

Laetmogonidae was an obvious polyphyletic group, and *Pannychiamoseleyi* Théel, 1882 was placed in the outmost clade of the other three families. *Laetmogonewyvillethomsoni* Théel, 1879 clustered with Elpidiidae and was sister to this clade; *Benthogoneabstrusa* Sluiter, 1901 was clustered with *Enypniasteseximia* Sluiter, 1901, but the Bayesian posterior probabilities and bootstrap values of this clade were low.

Based on the morphological and phylogenetic analyses, *B.jiaolongi* sp. nov. can be identified as a new species closely related to *B.sanguinolenta*. In addition, our specimens provided a new record of *P.verrucicaudatus* in the Western Pacific, broadening its distribution. Our results support the hypothesis that *Benthodytes* is paraphyletic and that the clade of *B.sanguinolenta* and *B.jiaolongi* sp. nov. is separated from the other species of *Benthodytes*.

## Supplementary Material

XML Treatment for Benthodytes

XML Treatment for Benthodytes jiaolongi

XML Treatment for Psychropotes verrucicaudatus

## References

[B1] Belyaev M Vinigradov E (1969) A new pelagic holothurian (Elasipoda, Psychropotidae) from abyssal depths in the Kurile-Kamchatka Trench. Zoologicheskii Zhurnal 48(5): 709–716.

[B2] Darriba D Taboada GL Doallo R (2012) jModelTest 2: more models, new heuristics and parallel computing. Nature Methods 9(8): 1–2. 10.1038/nmeth.2109 22847109 PMC4594756

[B3] Gebruk AV Kremenetskaia A Rouse GW (2019) A group of species “*Psychropoteslongicauda*” (Psychropotidae, Elasipodida, Holothuroidea) from the Kurile-Kamchatka trench area (North-West Pacific). Progress in Oceanography 180(2020): e102222. 10.1016/j.pocean.2019.102222

[B4] Hansen B (1975) Scientific results of the Danish deep-sea expedition round the world 1950–52. Systematics and biology of the deep-sea holothurians. Part. l. Elasipoda. Vinderup: The Galathea Committee, 1–262.

[B5] Hérouard E (1909) Triconus, nouveau genre de la famille des Psychropotineae. Bulletin Institut Musée Oceanographique, Monaco 145: 1–5.

[B6] Huelsenbeck JP Ronquist F (2001) MRBAYES: Bayesian inference of phylogenetic trees. Bioinformatics 17(8): 754–755. 10.1093/bioinformatics/17.8.754 11524383

[B7] Katoh K Standley DM (2013) MAFFT multiple sequence alignment software version 7: improvements in performance and usability. Molecular Biology and Evolution 30(4): 772–780. 10.1093/molbev/mst010 23329690 PMC3603318

[B8] Li Y-N Xiao N Zhang L-P Zhang H-B (2018) *Benthodytesmarianensis*, a new species of abyssal elasipodid sea cucumbers (Elasipodida: Psychropotidae) from the Mariana Trench area. Zootaxa 4462(3): 443–450. 10.11646/zootaxa.4462.3.10 30314039

[B9] Matthew L Richard M Amy W Steven S-H (2012) Geneious Basic: An Integrated and Extendable Desktop Software Platform for the Organization and Analysis of Sequence Data. Bioinformatics 28(12): 1647–1649. 10.1093/bioinformatics/bts199 22543367 PMC3371832

[B10] Miller AK Kerr AM Paulay G Reich M Wilson NG Carvajal JI Rouse GW (2017) Molecular phylogeny of extant Holothuroidea (Echinodermata). Molecular Phylogenetics and Evolution 111(1137): 110–131. 10.1016/j.ympev.2017.02.014 28263876

[B11] Perrier R (1896) Sur les Élasipodes recueillis par le Travailleur et le Talisman. Comptes rendus hebdomadaires des séances de l’Académie des sciences 123(21): 900–903.

[B12] P. Mark O’ L Paulay G Davey N Michonneau F (2011) The Antarctic region as a marine biodiversity hotspot for echinoderms: Diversity and diversification of sea cucumbers. Deep-Sea Research II 58(2011): 264–275. 10.1016/j.dsr2.2010.10.011

[B13] Rogacheva A Cross IA Billett DSM (2009) Psychropotid holothurians (Echinodermata: Holothuroidea: Elasipodida) collected at abyssal depths from around the Crozet Plateau in the Southern Indian Ocean. Zootaxa 2096(1): 460–478. 10.11646/zootaxa.2096.1.28

[B14] Silvestro D Michalak I (2012) raxmlGUI: a graphical front-end for RAxML. Organisms Diversity & Evolution 12(4): 335–337. 10.1007/s13127-011-0056-0

[B15] Stamatakis A (2014) RAxML Version 8: A tool for phylogenetic analysis and post-analysis of large phylogenies. Bioinformatics 30(9): 1312–1313. 10.1093/bioinformatics/btu033 24451623 PMC3998144

[B16] Takano T Itoh H Kano Y (2017) Dna-based identification of an echinoderm host for a deep-sea parasitic snail (Gastropoda: Eulimidae). Molluscan Research 38(3): 212–217. 10.1080/13235818.2017.1372865

[B17] Théel H (1882) Report on Holothurioidea. Pt. I. Report of the Scientific Results of the Voyage of H.M.S. Challenger. Zoology 4(13): 1–176.

[B18] Xiao N Gong L Kou Q Li X-Z (2019) *Psychropotesverrucicaudatus*, a new species of deep-sea holothurian (Echinodermata: Holothuroidea: Elasipodida: Psychropotidae) from a seamount in the South China Sea. Bulletin of Marine Science 95(3): 421–430. 10.5343/bms.2018.0041

[B19] Xiao N Li X Sha Z (2018) Psychropotid holothurians (Echinodermata: Holothuroidea: Elasipodida) of the tropical Western Pacific collected by the KEXUE expedition with description of one new species. Marine Biology Research 14(8): 816–826. 10.1080/17451000.2018.1546012

[B20] Zhang Z Bao X Dong Y Gao X Lei G Li S Liu W Hou H Shi J Pu H (2015) Complete mitochondrial genome of *Parastichopuscalifornicus* (Aspidochirotida: Stichopodidae). Mitochondrial DNA Part A 27(5): 3569–3570. 10.3109/19401736.2015.1074222 26260176

